# Emerging Therapeutic Potential of Polyphenols from *Geranium sanguineum* L. in Viral Infections, Including SARS-CoV-2

**DOI:** 10.3390/biom14010130

**Published:** 2024-01-19

**Authors:** Silviya Abarova, Ralitza Alexova, Stela Dragomanova, Ayten Solak, Paolo Fagone, Katia Mangano, Maria Cristina Petralia, Ferdinando Nicoletti, Reni Kalfin, Lyubka Tancheva

**Affiliations:** 1 Department of Medical Physics and Biophysics, Faculty of Medicine, Medical University of Sofia, Zdrave Str. 2, 1431 Sofia, Bulgaria; sabarova@medfac.mu-sofia.bg; 2 Department of Medical Chemistry and Biochemistry, Faculty of Medicine, Medical University of Sofia, Zdrave Str. 2, 1431 Sofia, Bulgaria; 3 Department of Pharmacology, Toxicology and Pharmacotherapy, Faculty of Pharmacy, Medical University of Varna, Tsar Osvoboditel Blvd 84A, 9002 Varna, Bulgaria; stela_dragomanova@abv.bg; 4 Institute of Cryobiology and Food Technologies, Cherni Vrah Blvd. 53, 1407 Sofia, Bulgaria; asolak@abv.bg; 5 Department of Biomedical and Biotechnological Sciences, University of Catania, Via S. Sofia 89, 95123 Catania, Italy; 6 Department of Clinical and Experimental Medicine, University of Messina, 98122 Messina, Italy; 7 Department of Biological Effects of Natural and Synthetic Substances, Bulgarian Academy of Sciences, Acad. Georgi Bonchev Str. 23, 1113 Sofia, Bulgaria; reni_kalfin@abv.bg (R.K.); lyubkatancheva@gmail.com (L.T.); 8 Department of Healthcare, South-West University “Neofit Rilski”, Ivan Mihailov Str. 66, 2700 Blagoevgrad, Bulgaria

**Keywords:** *Geranium sanguineum*, viral infection, COVID-19, poliphenols

## Abstract

The existing literature supports the anti-inflammatory, antioxidant, and antiviral capacities of the polyphenol extracts derived from *Geranium sanguineum* L. These extracts exhibit potential in hindering viral replication by inhibiting enzymes like DNA polymerase and reverse transcriptase. The antiviral properties of *G. sanguineum* L. seem to complement its immunomodulatory effects, contributing to infection resolution. While preclinical studies on *G. sanguineum* L. suggest its potential effectiveness against COVID-19, there is still a lack of clinical evidence. Therefore, the polyphenols extracted from this herb warrant further investigation as a potential alternative for preventing and treating COVID-19 infections.

## 1. SARS-CoV-2 and COVID-19

SARS-CoV-2 is a beta coronavirus responsible for the COVID-19 pandemic which, as of September 2023, has caused over 770 million cumulative cases and over 6.9 million deaths (www.covid19.who.int; accessed on 8 November 2023). The virus recognizes angiotensin-converting enzyme 2 (ACE2) on the surface of host cells. This receptor is expressed on many human cells, including lung epithelia, kidney, and cardiomyocytes and explains the pathology of COVID-19 as a multi-organ disease with the upper respiratory tract as a primary target.

After ACE2 binding, the viral spike (S) glycoprotein requires proteolytic processing in order to enter the host cells. Cathepsin L is an endosomal cysteine protease needed to prime the S protein. Knockdown of cathepsin L in the lungs was successful in reducing experimental SARS-CoV-2 infectivity in a mouse model, and alleviated brain pathology as well [1]. TMPRSS2 is a serine protease present on the lung cell membrane which can cleave the spike protein and facilitate fusion with the host cell [2]. In addition to the host proteases, the virus also contains two cysteine proteases: the main protease (M^pro^ or 3CL^pro^) and a papain-like protease (PL^Pro^). These proteases play a crucial role in the processing of the viral polyprotein, which is essential for the virus’s replication and lifecycle. Several monoclonal antibodies targeting the S protein (such as Bebtelovimab, Bamlanivimab, Etesevimab, Sotrovimab, Casirivimab, Imdevvimab, Regdanvimab, Tixagevimab, and Cilgavimab) have been approved by both the FDA and EMA for COVID-19 treatment. These antibodies work by preventing the virus from entering host cells.

Furthermore, various agents have been developed to inhibit viral replication. These include remdesivir, which acts as an inhibitor of the viral RNA polymerase; nirmatrelvir, an inhibitor of the viral main protease 3CLpro, typically used in combination with ritonavir; and molnupiravir, a nucleoside analog that induces mutations in the virus.

A third category of drugs for COVID-19 treatment focuses on modulating the host immune response to prevent severe organ damage caused by the hyperactivation of the immune system. These drugs include vilobelimab, which sequesters complement C5a; baricitinib, a Janus kinase inhibitor; tocilizumab, which binds to the IL-6 receptor; and anakinra, an antagonist of the IL-1 receptor. Notably, the latter three drugs have previously been employed to manage inflammation in rheumatoid arthritis. Long COVID, also known as post-acute sequelae of SARS-CoV-2 infection (PASC), is a complex and often debilitating condition that can affect individuals who have recovered from the acute phase of COVID-19. While the acute phase of the disease primarily involves respiratory symptoms, Long COVID encompasses a wide range of persistent and often unpredictable symptoms, extending far beyond the initial infection. These symptoms can affect various organ systems, including the respiratory, cardiovascular, neurological, and immune systems, and can significantly impact an individual’s quality of life. Long COVID remains an active area of research, and its full scope and underlying mechanisms are still being explored, making it a crucial focus in understanding the long-term consequences of the COVID-19 pandemic [3,4,5,6,7,8,9].

The persistent changes that COVID-19 induces in the body months after initial infection necessitate the availability of supportive therapies, which replenish the host’s defense systems and are non-toxic with long-term application. The novel SARS-CoV-2 mechanism in causing acute and Long COVID and emerging as a multi-organ dysfunction has spiked research activity in re-evaluating medicinal plants as a source for new pharmaceuticals. These efforts started early in the pandemic and have continued to the present day. Traditional medicine is a rich resource of knowledge on bioactive plant metabolites with pluripotent effects. Natural products often combine with antioxidant, antiviral and immunomodulatory effects [10]. Here, we review the anti-COVID-19 potential of polyphenols from *Geranium sanguineum* L., a herb used in Bulgarian traditional medicine.

## 2. *Geranium sanguineum* L. in Traditional Medicine

**Figure 1:**
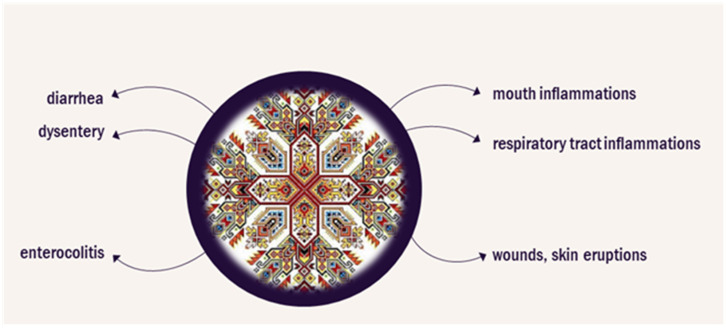
Application of *Geranium sanguineum* in Bulgarian traditional medicine.

Bloody cranesbill (*Geranium sanguineum* L.) is a flowering perennial herb with a natural range extending over Europe [11]. The herb is used in the ethnopharmacological practice in Bulgaria [12]. Figure 1 presents plant efficacy according to the Bulgarian traditional medicine in various disorders. Infusions and decoctions from the roots and aerial parts can be used as a rinse for inflamed mucous membranes of the mouth and respiratory tract, a wash for wounds or skin eruptions, for its astringent, anti-inflammatory, antiviral, hypotensive, and immunostimulant activity, as well as for supportive treatment of diarrhea, dysentery, and enterocolitis [12,13,14]. The extracts have an antibacterial effect against *S. aureus*, *E. coli*, *E. faecalis*, *K. pneumoniae*, *P. aeruginosa*, and *B. subtilis* [14,15,16]. A polysaccharide extracted from the roots inhibits the growth of *S. enterica* [17]. The antibacterial properties of the essential oil from the flowers and the aerial parts of the herb have also been explored and more than 240 chemical components in the extracts have been identified [18,19]. An ethanol extract containing mainly anthocyanidins from the roots was reported to have antitumor activity in vitro and in a murine model of Ehrlich’s breast carcinoma [20].

An early survey of the Geranium spp. showed that *G. sanguineum* was among species with high ellagitannin content in the leaves [21]. The main polyphenol component according to Mavlyanov et al. [22] is bis-hexahydroxydiphenoyl-trigalloylglucose, similar to the condensed tannin from *Geranium thunbergii*.

The plant shares relatively high quercetin levels with other *Geranium* spp. but its content of myricetin is distinctive and not wide-spread in the genus [11]. This was more recently confirmed by Ivancheva and Petrova [23] who found myricetin, quercetin (including quercetin-3-glucoside and -galactoside), and kempferol. Whole-plant extracts identified quercetin and kempferol, as well as their glycosides (quercitrin, isoquercitrin, hyperoside, rutin), caftaric, and caffeic acid [24]. A combination of extraction methods achieved the identification of flavonoids, phenolic acids, and hydrolysable tannins including gallic acid, kaempferol, quercetin, rutin, 2-galloylglucose, 3-galloylglucose, and 2,3-digalloylglucose [22]. In the roots, polyphenols of the condensed type were dominant, including (+) catechin, (+/−) gallocatechin, and three protoanthocyanidins [25].

Another comparison of eight *Geranium* spp. detected the highest concentration of polyphenols and tannin content in *G. sanguineum* aerial parts [15]. While the amount of polyphenols in the leaves is 9–11%, it is even higher in the roots, at up to 18% [22]. Similar values for the total phenolics content in the leaves were obtained in a study by Maslennikov et al. [26], who identified *G. sanguineum* as the second most phenol-rich plant among 66 species of plants included in the study.

The later part of the 20th century saw systematic attempts to characterize the active components present in a standardized polyphenol complex (PPC) of *G. sanguineum* L. This extract from aerial roots yields a dark red water-soluble powder on lyophilization and contains 34.6% (*w*/*w*) total soluble phenolics with 16.15% represented by tannins, 0.126% flavonoids and 2.12 mg/kg catechins and proanthocyanidins [27,28,29,30]. Slightly higher values for tannins (19.7%) and flavonoids (0.22%) were obtained by Benzel et al. [14].

The standardized PPC extract contains caffeic acid, gallotannin, (+/−), catechin, (−) epicatechin, quercetin, hyperoside, apigenin, myricetin, morin, maltol, and additional unidentified flavonoids [29,30]. Thin-layer chromatography identified ellagic acid in the extract, but this was reported only in some later studies [30,31].

**Figure 2:**
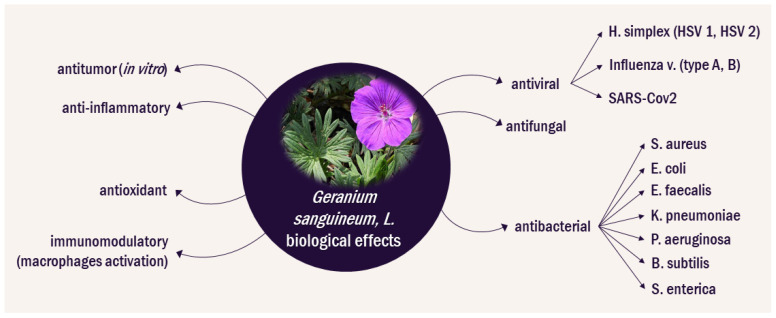
Documented biological effects of *Geranium sanguineum* [10,12,13,14,15,16,17,18,19,20,28,30,31,33,34,35,36,37,38,39,40,41,42,43,44].

More recently, the polysaccharide components of *G. sanguineum* L. extracts have also been analyzed. The total polysaccharide content in leaves has been reported to be 27% (*w*/*w*) and in roots 56.8% (*w*/*w*) [17]. The lectin content in the roots of *G. sanguineum* L. is high, exceeding that of *G. robertianum* or *G. sibiricum*, but further studies on the lectin composition of the herb are lacking [32]. Documented biological effects of *G. sanguineum* extracts are summarized in Figure 2.

The standardized PPC from *G.sanguineum* L. has raised interest because of its activity against herpes and influenza virus both in vitro, as well as in in vivo rodent models [10]. The availability of a standardized extract has allowed for the mechanism of its bioactivity to be explored in a controlled fashion. There is an apparent strong synergy between the components of the extract. In addition, the extract seems to be not only directly antiviral, but also to be capable of modulating the oxidative and immune environment. Several studies on the *G. sanguineum* L. extracts have been conducted before the emergence of the SARS-CoV-2 pandemic. We will now explore these studies within the context of the potential applications of *G. sanguineum* L. extract for the supportive treatment of both COVID-19 and its long-term consequences, known as Long COVID. This review seeks to shed light on how the findings from these earlier investigations may contribute to our understanding and management of the critical aspects of the past global health crisis, due to COVID-19 pandemic.

## 3. Antiviral Activity of *G. sanguineum* L. Polyphenol Complex

Both herpes simplex virus HSV1 and HSV2 and influenza virus type A and B have been shown to be susceptible to treatment with PPC [30,33]. In vitro, the extract was effective if applied within 3 h post-infection [45]. In the case of herpes infection, inhibition was more effective if PPC was applied after inoculation with the virus than as a pretreatment [33]. The main effects of *G. sanguineum* L. against influenza infection are summarized in Table 1.

For the influenza infection rodent model, among the several different application routes tested, the aerosol PPC was found to be effective [31,34,35]. Intranasal application in mice at 10 mg/kg 3 h prior to virus exposure was effective in vivo against human influenza A/Aichi (H_3_N_2_) [10,27]. The treatment 24 h before infection was the most effective, but it also worked if it coincided with the viral inoculation [31]. The benefits of PPC intranasal delivery show promise for the prophylaxis of other infections of the respiratory tract, including those caused by the coronaviridae. The advantage of this mode of application is that it does not depend on the metabolism of the ingested polyphenols, which are generally poorly bioavailable and extensively metabolized when taken orally.

Administration of the PPC decreased viral loads in mice, reduced the extent of the lung lesions, and increased mean survival time in mice. Accordingly, PPC induced normalization of the protease activity levels in the lung of the infected mice [36].

Combined with a protease inhibitor extract from *Streptomyces* sp. 225b, the polyphenol complex of *G. Sanguineum* L. reduced the replication of influenza virus in MDCK cells [34,46]. The PPC also showed synergistic effects with ε-aminocaproic acid (a serine protease inhibitor) when used in experimentally induced viral infection in mice [47]. In vitro, the PPC alone inhibited dose-dependently several proteases including trypsin, pepsin, proteinase K, and cathepsin, but not subtilisin or chymotrypsin [36].

The PPC may also prevent viral replication by targeting polymerases. A preparation from the epigeal parts of the plant showed inhibition of DNA polymerase and reverse transcriptase, although it was not active in inhibiting RNA polymerase. [22]

The ability of the PPC to inhibit the development of lesions from HSV, as well as the traditional use of the *G. sanguineum* L. herb in topical treatment for skin conditions, including the oral mucosa, makes it worthwhile to explore the properties of the *G. sanguineum* L. PPC extract in Long COVID with dermatological involvement. In contrast to influenza infection, the effect on *G. sanguineum* L. on other viruses has not been explored as systematically.

## 4. Antioxidant Effects of *G. sanguineum* L. Polyphenol Complex

A driver of tissue damage in viral infection which seems to be targeted by the *G. sanguineum* L. PPC is oxidative stress. During an immune response, activated cells, such as neutrophils and macrophages, are able to launch an attack on microbes and signal to other cells via the production of reactive oxygen and nitrogen species (RONS). Oxidative stress is a downstream effect of SARS-CoV-2, binding to ACE2 and triggering of NADPH oxidases [48].

There are correlations between high levels of TBARS (lipid peroxidation products) in the lungs, as well as in the blood and liver, and the inhibition of hepatic CYP-450, responsible for the metabolism of many drugs and endogenous compounds. Data from our group have indeed shown a significant negative correlation between the increased TBARS levels and the decreased content of cytochrome P450 in infected animals [10,27,49].

The prophylactic and therapeutic role of several natural antioxidants, vitamins, and polyphenols on experimental models of viral infections has been extensively reviewed in previous publications [10,50]. Polyphenols increase the antioxidant capacity of tissues by increasing SOD and GPx levels, as well as create microenvironments with reduced ROS [51]. Along the same lines, the antioxidant capacity of extract from *G. sanguineum* L. has been already observed in vivo, in influenza-infected mice [37,38].

In a comparative panel assessing the free radical scavenging activity of various *Geranium* spp., the aerial parts of *G. sanguineum* L. demonstrated exceptional antioxidant capabilities, second only to *G. macrorhizum* [52]. In another study comparing different Geranium species, it was revealed that the aerial parts of *G. sanguineum* L. exhibited the second-highest total antioxidant activity, closely following *G. palustre*, and this robust activity corresponded to its high polyphenol content [15].

The PPC complex effectively inhibited the production of superoxide (O_2_^−^) radicals [39]. Both the PPC and the fraction extracted with EtOAc exhibited strong superoxide scavenging capabilities, synergizing with the presence of SOD in the test environment [40]. When employed to support an exogenous SOD from Humicula lutea, the *G. sanguineum* extract displayed a synergistic response, even at doses that are typically considered too low (up to 8 times less) to have a significant effect on their own [53].

Hepatic P-450 mono-oxygenases are membrane-bound enzymes that can be inhibited by viral infections, such as influenza. Our research group demonstrated that a standardized *G. sanguineum* PPC effectively restored the activity of these enzymes [10,27]. The inhibition of these liver enzymes during the disease process seems to result from the generation of free radicals. Therefore, it was hypothesized that the mechanism by which pretreatment with PPC alleviates enzyme inhibition is through its antioxidant properties, as evidenced by decreased levels of TBARS and TAA [10,27].

The pro-oxidant effects in healthy mice and the antioxidant properties in virus-infected mice of a polyphenol-rich extract from *G. sanguineum* L. were confirmed through both in vitro and in vivo studies. In hepatocytes, membrane lipid peroxidation induced by Fe^2+^ ascorbate, as measured by malonyl dialdehyde (MDA), decreased in a dose-dependent manner with PPC treatment, with significant effects observed at concentrations up to 25 µg/mL [41].

Oxidized phospholipids in the lungs play a crucial role in COVID-related injuries, as they are associated with the activation of endothelial cells for monocyte recruitment and macrophage activation (as reviewed in [48]).

In the context of COVID-19, a multi-omics study has reported alterations in the ferroptosis pathway and lipid metabolism in erythrocytes of infected patients [54]. In particular, it was found that the red blood cells of COVID-19 patients contained lower levels of SOD1 and higher levels of oxidized glutathione [54].

The alterations in the erythrocyte membrane appear to be linked to the persistent reduction in size and deformability of erythrocytes in COVID-19 patients, even months after recovery [9]. It is possible that bolstering the endogenous antioxidant systems with natural products like *G. sanguineum* PPC could offer significant benefits. The PPC exhibited a dose-dependent capacity to prevent increases in the permeability of erythrocyte membranes, without impacting catalase activity [41]. Furthermore, no notable effects were observed on H_2_O_2_ generation at physiological pH or on catalase activity [39,41].

The PPC complex also exhibits chelating properties for iron [39]. Elevated levels of IL-6 in COVID-19 can lead to increased ferritin and hepcidin levels. Hepcidin plays a role in storing iron in macrophages, potentially impacting the iron pool available for generating reactive oxygen species (ROS) and ferroptosis [48].

In alveolar macrophages, the combined treatment decreased superoxide and hydrogen peroxide levels and restored the activity of SOD and CAT enzymes [53]. The extract alone was also effective in restoring their enzymatic activity [46]. Furthermore, the presence of PPC resulted in decreased levels of H_2_O_2_, O_2_^−^, and NO produced by alveolar macrophages during influenza infection [35,42]. In healthy mice, spontaneous, though not inducible, NO production by peritoneal macrophages was also reduced [55].

Catechol and galloyl-containing polyphenols can exhibit pro-oxidant properties as they scavenge free radicals from semi-quinones and reduce Fe^3+^ [51]. The PPC complex displayed pro-oxidant effects on lung membrane lipids in intact mice. However, in the influenza infection model, the *G. sanguineum* L. extract acted as an antioxidant, effectively returning elevated levels of malonyl dialdehyde (MDA) to control levels [10,27,41]. A mild pro-oxidant effect was also observed with regard to CYP450 content and aniline hydroxylase activity in healthy animals [27]. In healthy mice, the presence of PPC led to an increase in alveolar macrophage superoxide production [34,36]. During the early stages of the infection (day 2), PPC further augmented superoxide production in alveolar macrophages [34,36]. Notably, the butanol (BuOH) fraction of the PPC demonstrated superoxide-generating effects and contained the majority of the antiviral effect in vitro [40]. This suggests that reactive oxygen species generation early in the viral infection is an important mechanism for the *G. sanguineium* PPC to inhibit viral replication [40].

## 5. Immunomodulatory Effects

Numerous studies have highlighted the distinctive mode of action exhibited by the PPC extract in both in vivo and in vitro settings. This divergence may arise from its capacity to influence immune signaling within the broader context of the whole organism. Unlike the hydrolysable tannins, this variance is unlikely to result from substantial metabolic transformations of the polyphenols within the extract. Instead, it is probable that when *G. sanguineum* is topically applied in the context of viral infections, it predominantly elicits a localized response.

Macrophages are the most abundant immune cell in the lung and an increase in macrophages and transcript of matrix metalloproteases (MMPs) has been observed in the lungs of COVID-19 patients [2,56,57]. Alveolar macrophages secrete chemokines and cytokines, which recruit monocytes and neutrophils and lead to tissue damage [4]. In severe COVID-19, fibrotic remodeling of the lung is characteristic [4]. Monocyte-derived macrophages are important for the response and are directed to the lungs by the release of chemokines from lung pneumocytes. This occurs on both acute and post-acute stages of the disease [4]. Activated macrophages also lead to endothelial involvement and coagulation disorders associated with severe COVID-19 [4].

Tissue damage in viral disease is often caused by the deregulated action of immune cells. An immune response is necessary to promote survival and decrease viral loads, but this needs to be counterbalanced by the ability of a deregulated inflammatory response to cause immunopathology [57]. In the lung, resident and recruited macrophages can drive tissue remodeling, granulocyte infiltration, and airway inflammation [8]. Type-I IFN is necessary to control IL-6 levels and macrophage activity and to minimize tissue damage, including in respiratory infections [57]. In COVID-19, the immune system mounts an exaggerated chemokine and interferon response which persists in the macrophage population several months after initial infection. In severe COVID-19, patients had reduced type I IFN responses and CD68+ macrophage infiltration in the lung [57].

The effect of PPC has not been tested in the context of SARS-CoV-2 infection, but several studies have been performed in influenza models. Pretreatment with PPC extract from *G. sanguineum* decreased the lung damage in mice and extended their mean survival time [28,31,36,42]. Lung pathology was independent of influenza viral titers [57]. Thus, the direct antiviral effects of the PPC extract may be synergistic to its immunomodulatory properties in mounting an appropriate immune response and leading to resolution of the infection.

In the extracts analyzed by Georgiev et al. [17], the total phenolic content was low relative to the polysaccharides. Both human monocytes and granulocytes could be stimulated with the plant extract. The root extract was able to expand the CD69+-activated population and induced release of IL-6 from macrophages. Even though the extract contained some phenolic compounds, its antioxidant activity was low, suggesting that the immunomodulatory action of *G. sanguineum* extracts can be dissociated from their antioxidant effects. Similarly, to the dual pro- and antioxidant function of the PPC, the authors pointed out that polysaccharides can be immunostimulatory but in an inflammation context can have anti-inflammatory activity [17].

In healthy and influenza-infected mice, the polyphenol extract caused an increase in the number of peritoneal and alveolar macrophages, as well as an increase in their migration and in phagocytic activity [28,36,42]. The increase in numbers was the highest in healthy alveolar macrophages exposed to the extract [28]. Phagocytosis by blood PMNs also increased [28]. In vitro, at 12.5 and 25 µg/mL, the extract did not have a significant effect on migration of alveolar or peritoneal macrophages [42,55].

## 6. Effects of Individual Ingredients in the *G. sanguineum* Polyphenol Extract

One of the challenges encountered in the study of natural products is unraveling the synergistic effects resulting from the components within the extract. Frequently, when purified components are individually tested, a loss of activity is observed, making it difficult to provide a detailed mechanistic explanation for the observed bioactivity. This challenge is also evident in the context of the *G. sanguineum* extract, where the ethyl acetate and butanol fractions show distinct antioxidant and antiviral properties. The disparities observed may arise from variations in the composition or concentration of individual components within the fractions, highlighting that individual components do not produce identical effects [40].

High concentrations of phenolic compounds may be present, but they could exhibit limited bioavailability, thus explaining the differences between their in vitro and in vivo effects. Indeed, pure substances such as myricetin, (−) epicatechin, and (+/−) catechin do not exhibit the same antiviral effect as the extracts containing them [29]. Notably, myricetin, found in the ethyl acetate fraction, demonstrated an EC_50_ closest to that of the whole extract [29]. This is significant as myricetin, a component less abundant in other members of the genus Geranium, may contribute to the therapeutic effects characteristic of *G. sanguineum*.

In the specific context of myricetin, its incubation with Vero E6 cells inhibited SARS-CoV-2 replication, despite its high hydrophilicity, which would normally reduce its ability to enter cells [43,44,58]. Additionally, myricetin interfered dose-dependently with the binding of the S protein to ACE2 expressed by HEK293 cells [44]. Treatment with myricetin also demonstrated a reduction in hyperinflammation markers, such as RIPK and NFkBp65 phosphorylation, in LPS-stimulated macrophages [44]. In a mouse model, myricetin showed a protective effect against acute lung injury, reducing lung edema and alveolar inflammation [44]. Furthermore, myricetin reduced inflammatory cell counts in the BALF of bleomycin-treated mice and normalized levels of inflammatory markers [59].

Notably, myricetin glycoside and rhamnoside (myricitrin) from *Camellia sinensis* and *Myrica cerifera*, respectively, demonstrated binding affinities against the 3CL^Pro^ viral protease of SARS-CoV-2 in silico [60]. Myricetin itself exhibited covalent binding to the catalytic cysteine of SARS-CoV-2 and SARS-CoV main protease 3CL^Pro^, with weaker affinity for PL^Pro^ [43]. This binding could occur after myricetin autooxidation, suggesting a potential benefit in a pro-oxidative cellular environment during early viral infection in the upper respiratory tract or in the presence of activated immune cells [43].

In the whole extract of *G. sanguineum*, myricetin activity may be complemented by the antiviral properties of a non-identified gallotannin, caffeic acid, catechin, and epicatechin [29]. Quercetin and quercetin-O-galactoside, present in the extract, also showed similar EC_50_ values to the whole extract, indicating their potential contribution [31,36]. Incubation with quercetin and catechin demonstrated inhibitory activity against various viruses [61], and in vivo studies showed the preventive and restoring effects of rutin and quercetin in a mouse influenza virus infection model [50].

Moreover, quercetin displayed inhibitory activity towards the main protease of SARS-CoV-2, suggesting its potential synergy with kempferol, both present in *G. sanguineum* extract [62]. Importantly, these polyphenols did not alter the secondary structure of the enzyme [62]. These findings collectively emphasize the complex interplay of components within natural extracts, the nuanced effects of individual compounds, and the potential for synergistic actions in the pursuit of antiviral therapeutic agents. Table 2 summarizes the molecular mechanisms of the main identified compounds of *G. sanguineum* L. and their action against SARS-CoV-2 [62,63,64,65,66,67,68,69,70,71,72,73,74,75,76,77,78,79,80,81,82,83,84,85,86,87,88,89,90,91,92,93,94,95,96,97,98,99].

## 7. Conclusions

This extensive review of the available literature strongly underscores the therapeutic potential of polyphenol extracts derived from *G. sanguineum* L. These polyphenols exhibit significant anti-inflammatory, antioxidant, and antiviral properties, establishing *G. sanguineum* L. as a noteworthy candidate in the field of natural remedies.

One of the distinctive characteristics of *G. sanguineum* L. is its capacity to impede viral replication by inhibiting DNA polymerase and reverse transcriptase. This direct antiviral effect couples synergistically with its immunomodulatory properties, contributing to the resolution of infections. Specifically, the prevalence of condensed tannins in *G. sanguineum* L. emerges as a pivotal factor in mitigating lung damage during respiratory viral infections, including the potential implications for COVID-19. These tannins demonstrate efficacy by inhibiting pulmonary protease activity and modulating macrophage responses, thereby offering a multifaceted defense against respiratory viruses.

Furthermore, the antioxidant properties inherent in the polyphenols extracted from *G. sanguineum* L. play a crucial role in elevating tissue antioxidant capacity. This elevation is achieved by upregulating levels of superoxide dismutase (SOD) and glutathione peroxidase (GPx), creating a microenvironment with reduced reactive oxygen species (ROS). An additional vital mechanism involves the restoration of the activities of hepatic P-450 mono-oxygenases, enzymes typically inhibited by viral infections.

While preclinical studies using *G. sanguineum* L. suggest its promising effectiveness against COVID-19, the current state of clinical evidence is still limited. It is imperative to acknowledge this gap and emphasize the need for further research to substantiate the potential of the polyphenols derived from *G. sanguineum* L. as a viable alternative for the prevention and treatment of COVID-19 infections. Continued exploration in both preclinical and clinical settings will be instrumental in unlocking the full therapeutic potential of this natural resource.

In conclusion, *G. sanguineum* L. emerges as a promising avenue for future research, with its polyphenols presenting a multifaceted approach in combating inflammation, oxidative stress, and viral infections, especially in the context of respiratory diseases like COVID-19.

## Figures and Tables

**Table 1:** Effects of *Geranium sanguineum* L. antiviral action against influenza infection.

Mechanism	Type of Experiment	References
Decreased viral load	mice	[36]
Reduced extent of lung lesions	mice	[36]
Normalization of lung protease activity	mice	[36]
Reduced replication (in combination with protease inhibitor from *Streptomyces* spp.)	MDCK cells	[34,46]
Inhibition of trypsin, pepsin, proteinase K, and cathepsin	in vitro	[36]
DNA polymerase inhibition	in vitro, in ovo	[22]
Reverse transcriptase inhibition	in vitro, in ovo	[22]
Increased SOD activity	mice	[37,38]
Increased GPx levels	mice	[37,38]
Decreased levels of H_2_O_2_, O_2_, and NO produced by alveolar macrophages	mice	[35,42]
Decreased MDA levels	mice	[10,27,41]
Superoxide generation in early stages of viral infections	in vitro	[40]
Increased number of peritoneal and alveolar macrophages	mice	[28,36,42]
Stimulated migration and phagocytic activity of macrophages	mice	[28,36,42]

**Table 2:** Effects of *Geranium sanguineum* L. in regard to SARS-CoV-2 infection.

Content of *Geranium sanguineum* L.	Type of Study	Main Anti-COVID Effects	References
Quercetin 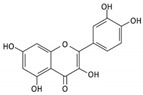	Case-control clinical studies	Multifactor beneficial action against SARS-CoV-2	[63]
		Several stages of the viral life cycle inhibition (from entry to replication of SARS-CoV-2)	[64,65,66,67,68,69,70]
		Glycoprotein spike direct binding, ACE2 activity inhibition with disrupting the viral–host recognition interface, and preventing the SARS-CoV-2 entry	[71]
		Alter the expression of several human genes encoding protein targets of SARS-CoV-2, thus potentially interfering with the functions of the viral proteins in human cells	[63]
		Viral replication inhibition by interfering with the activity of 3-chymotrypsin-like protease (3CL^pro^), papain-like protease (PL^pro^), and RNA-dependent RNA polymerase (RdRp)	[63]
		Antioxidant, anti-inflammatory, and immunomodulation actions contributing to mitigating the disease consequences	[72,73,74,75,76]
		SIRT1/NLR3 pathway (SIRT1 promotion) modulation	[77]
		Inflammasome (TXNIP3 inhibition)	[78]
		NLRP3 inflammasome components (NLRP3, ASC, activated caspase-1 inhibition)	[79]
		TH17/Treg modulation:	[80]
		Treg-related cytokine activation;	
		TH17-related cytokine inhibition;	
		TH17/Treg inhibition	
		Proinflammatory cytokines and inflammatory mediators’ inhibition	[80]
		IL-1β, IL18, TNF-α, IL6, PGE2, COX-2, and i-NOS inhibition	[81,82,83,84]
Myricetin 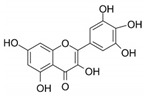	In silico and in vitro studies	Coronavirus entry and replication cycle inhibition due its interaction with s-protein and ACE-2, M^pro^, PL^pro^, helicase, exonuclease, and endoribonuclease	[85]
		A unique mode of covalent bonding of MYR in targeting the M^pro^ responsible for suppression of enzyme activity	
		Inflammatory and immune processes modulation	
Apigenin 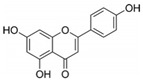	LigPlot analysis, In-silico Osiris/Molinspiration and ADMET analysis	M^pro^ SARS-CoV-2 inhibition	[86]
Kaempferol 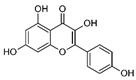	Bioinformatics and computational results	Modulation of pathways/targets related to inflammation, immune regulation, and viral infection	[87]
Catechin 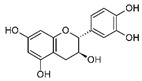	Molecular docking	Best affinity towards the spike proteins from all the catechins (higher than the native ligand)	[88]
Chlorogenic acid 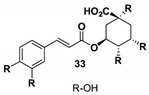	Network pharmacology, followed by molecular docking	Inflammatory response in COVID-19 modulation	[89]
		Integrating three common receptors of SARS-CoV-2	
Caffeic acid 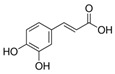	Molecular docking	Modulation of COVID-19 with higher binding energies than nelfinavir against COVID-19 M^pro^, Nsp15, SARS-CoV-2 spike S2 subunit, spike open state and closed state structure, respectively	[90]
Epicatechin 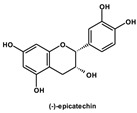	Molecular dynamics simulations	Static and dynamic inhibition for ACE2 with highly favorable pharmacokinetic properties than the Other known ACE2 inhibiting compounds	[91]
		Blocking/weakening the SARS-CoV-2 entry and its subsequent invasion	
Rutin 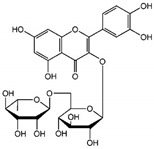	Molecular dynamics simulation and docking studies	Matched very well with the 6GLU7 binding pocket	[92]
		A potential inhibitor of M^pro^	
Hyperoside 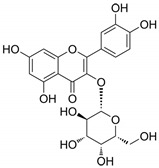	Elucidation of human and mouse macrophages	NLRP3 inflammasome inhibition	[93]
		AIM2 and NLRC4 inflammasomes activation impairment	
		Caspase-1 activity inhibition	
Tannins 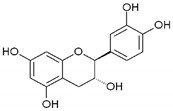	FRET-based enzyme activity assay of M^pro^; Surface plasmon resonance analysis; Molecular docking; Vpp pseudoviral particle infection assay	Dual inhibition of TMPRSS2 and M^pro^/3CL^pro^ with subsequent SARS-CoV-2 activity inhibition	[94,95]
	Docking characterization and in vitro Inhibitory activity	Dual inhibition of TMPRSS2 and M^pro^/3CL^pro^ with suppression of cellular entry of the virus	[94]
		High potential for SARS-CoV-2 inhibition	[96,97,98,99]
